# Che-1/AATF: A Critical Cofactor for Both Wild-Type- and Mutant-p53 Proteins

**DOI:** 10.3389/fonc.2016.00034

**Published:** 2016-02-15

**Authors:** Tiziana Bruno, Simona Iezzi, Maurizio Fanciulli

**Affiliations:** ^1^SAFU Laboratory, Department of Research, Advanced Diagnostic, and Technological Innovation, Regina Elena Cancer Institute, Rome, Italy

**Keywords:** Che-1/AATF, p53, apoptosis, survival

## Abstract

The p53 protein is a key player in a wide range of protein networks that allow the state of “good health” of the cell. Not surprisingly, mutations of the TP53 gene are one of the most common alterations associated to cancer cells. Mutated forms of p53 (mtp53) not only lose the ability to protect the integrity of the genetic heritage of the cell but also acquire pro-oncogenic functions, behaving like dangerous accelerators of transformation and tumor progression. In recent years, many studies focused on investigating possible strategies aiming to counteract this mutant p53 “gain of function” but the results have not always been satisfactory. Che-1/AATF is a nuclear protein that binds to RNA polymerase II and plays a role in multiple fundamental processes, including control of transcription, cell cycle regulation, DNA damage response, and apoptosis. Several studies showed Che-1/AATF as an important endogenous regulator of p53 expression and activity in a variety of biological processes. Notably, this same regulation was more recently observed also on mtp53. The depletion of Che-1/AATF strongly reduces the expression of mutant p53 in several tumors *in vitro* and *in vivo*, making the cells an easier target for chemotherapy treatments. In this mini review, we report an overview of Che-1/AATF functions and discuss a possible role of Che-1/AATF in cancer therapy, with particular regard to its action on p53/mtp53.

The *TP53* gene is a tumor suppressor capable of detecting oncogenic events in tumor cells and eliminating them through using several different mechanisms. It is the most frequently mutated gene in human cancers, and p53 mutant forms (mtp53), in addition to losing the function of the wild-type p53 as “guardian of the genome,” acquire specific properties that contribute to the aggressiveness and chemoresistance of cancer (1). The activity of wild-type p53 is modulated through various mechanisms, which contribute to its full functionality, regulating both its stability and its specificity of action. Notably, these same mechanisms also operate on mtp53, sustaining its oncogenic functions (2–4). Che-1/AATF was recently identified among the proteins that can not only regulate p53 functions but also support the activity of oncogenic mtp53. In this mini review, we provide an updated overview of Che-1/AATF activities, detailing its intimate connection with p53.

## Che-1/AATF

Che-1/AATF was identified in the early years of this decade by different groups both as a protein involved in the control of transcription and apoptosis, and a gene downregulated upon TGFβ induced differentiation (5–8). This protein is able to connect specific transcription factors to the general transcriptional machinery through its interaction with the subunit 11 of RNA polymerase II (hRPB11) (6). In particular, Che-1/AATF has been shown to interact and modulate the activity of several nuclear hormone receptors (9) and transcription factors, including the retinoblastoma protein (pRb), p65 and STAT3 (10–12). These interactions are mostly regulated by post-translational modifications, which provide a rapid and reversible manner to modulate Che-1/AATF co-transcriptional activity in response to different stimuli. Moreover, Che-1/AATF action on transcription may also be modulated by its binding to different forms of hRPB11. Indeed, this subunit is the product of a multigene family, which encodes specific proteins differently expressed in several tissues and showing different binding capacities (13, 14). Che-1/AATF protein is found expressed in all tissues (6, 7), and its expression is required for proliferation and survival. Indeed, Traube (Che-1/AATF mouse orthologous) knock out mice halt the development at the compacted morula stage and are embryonically lethal. Furthermore, mutant embryos exhibit a reduction in cellular proliferation (15), indicating Che-1/AATF’s involvement in cell cycle regulation. Consistent with these observations, Che-1/AATF has been shown to be involved in cell cycle progression through its ability to affect pRb protein’s growth suppression functions (10, 16). Moreover, it was demonstrated that Che-1/AATF localizes at interphase centrosomes and regulates centrosome duplication and spindle formation indicating a role for Che-1/AATF in the control of mitotic entry (17). Che-1/AATF not only regulates cellular proliferation but also has a significant role in controlling the apoptotic process. To date, most of the information regarding the antiapoptotic function of Che-1/AATF derives from studies performed in the neural tissue, where this protein appears to take part in regulating apoptotic activation in both physiological and pathological conditions (18–21). Moreover, Che-1/AATF interacts with cytoplasmic Tau in rat cerebellar granule neurons, and this interaction is modulated during neuronal apoptosis (22). A protective role of Che-1/AATF has also been described in human kidney proximal tubule cells, where this protein antagonizes apoptotic cell death by preserving mitochondrial function and reducing oxidative damage (23). Alternatively, Che-1/AATF has also been reported to have a pro-apoptotic role. Indeed, Che-1/AATF overexpression increases UV-induced apoptosis by promoting phosphorylation and transcriptional activity of the apoptotic gene c-Jun, in a p53 independent way. Moreover, UV damage induces Che-1/AATF redistribution from the nucleolus to the nucleoplasm, thus allowing Che-1/AATF and c-Jun to directly interact (24).

## Che-1/AATF within WT-p53 Tumor Suppressor Activities

The tumor suppressor p53 is one of the main effector of the DNA damage response (DDR), a complex network of pathways responsible for maintaining genome integrity and preventing tumorigenesis (25, 26). DDR coordinates several pathways that cooperate together to detect DNA lesions, arrest cell cycle in order to allow repair, and induce apoptosis or senescence if damage is too severe (27). p53 is a key signal integrator of these pathways, capable of regulating the transcription of a large variety of target genes, and for this reason, its levels and activities are tightly regulated inside the cell. Upon DNA damage, p53 expression can be enhanced at both transcriptional (28) and translational level (29). However, its functions are largely modulated by post-translational modifications and protein–protein interactions (30). In the last past years, several studies have identified Che-1/AATF as an important component of DDR and an endogenous p53 regulator (8). In response to genotoxic stress, Che-1/AATF is extensively modified by post-translational modifications affecting its localization, half-life, and interactions (8). Among these modifications, phosphorylation by checkpoint kinases ATM and Chk2 has a pivotal role in the context of Che-1/AATF-p53 connection. Indeed, this modification greatly affects Che-1/AATF functions, acting as a molecular switch that moves this protein from the pathways promoting cell cycle progression to the ones involved in cell cycle arrest and survival. In particular, (ATM–Chk2) phosphorylated-Che-1/AATF interacts with NF-kB p65 subunit, and this interaction moves Che-1/AATF from E2F1-dependent promoters to the *TP53* promoter, thus increasing transcription of this gene and contributing to the increase of p53 protein levels after genotoxic stress (11). Notably, phosphorylated-Che-1/AATF activates p53 transcription also in the absence of genotoxic stress, probably as a consequence of an intrinsic DNA damage occurring during DNA replication (11). This observation leads to hypothesize a model in which Che-1/AATF is already required for the basal state of p53 expression, and this activity is reinforced in response to DNA damage. Moreover, Che-1/AATF plays an important role in the maintenance of the G2/M checkpoint, and this effect depends on the activation of p53. Consistent with these findings, Che-1/AATF depletion was found to sensitize cancer but not normal cells to antineoplastic drugs (11).

In addition to sustaining *TP53* transcription, Che-1/AATF phosphorylation also promotes the binding of Che-1/AATF to p53, regulating in such way p53 activities (31). Of interest, Che-1/AATF is a component of a ternary complex with p53 and Brca1, and p53 is required for these interactions. This complex is observed at the early stage of the DDR, and when DNA damage is too extensive and cells undergo apoptosis, p53 modifications produced by Pin1 induce the detachment of the proteins. Notably, the interaction between Che-1/AATF-p53 specifically directs p53 toward the transcription of genes involved in growth arrest over its pro-apoptotic target genes. Indeed, a Chip-Seq analysis revealed a strong enrichment of p53 target genes involved in apoptosis in Che-1/AATF depleted cells, with a concomitant decrease in genes regulating growth arrest (31).

Höpker et al. have described another mechanism by which Che-1/AATF modulates p53 activity. They highlighted a cytoplasmic localization of Che-1/AATF in absence of DNA damage, and demonstrate that in response to genotoxic stress, this protein translocates from the cytoplasm to the nucleus, as a consequence of a phosphorylation by the checkpoint kinase MK2 (32). Remarkably, nuclear Che-1/AATF regulates the cellular outcome of the p53 response by competing with this protein for the binding to the promoter of several apoptotic genes, inhibiting in such way their activation (32).

Consistent with all these findings, Che-1/AATF^+/−^ mice exhibited a greater apoptosis in response to genotoxic stress when compared to wild-type littermates. Furthermore, thymocytes from Che-1/AATF^+/−^ mice showed an increase of p53 protein on pro-apoptotic gene promoters (31), thus confirming that Che-1/AATF controls p53 activity both *in vitro* and *in vivo*.

A further indication of the intimate relationship between Che-1/AATF and p53 arises from the observation that p53 binds the promoter of *Che-1/AATF* gene in response to DNA damage, leaving to assume the existence of a regulatory feedback loop between the two proteins (31). Moreover, there have been numerous findings that showed how many pathways operating on p53 are actually involved in Che-1/AATF regulation. In fact, the pro-apoptotic kinase HIPK2 phosphorylates Che-1/AATF at residue T144 in response to apoptotic DNA damage. This modification permits the prolyl isomerase Pin1 to produce a conformational change, facilitating the interaction with ubiquitin ligase HDM2, thereby inducing Che-1/AATF ubiquitylation and proteasomal degradation (33, 34). Notably, not only does Che-1/AATF activate the transcription of p53 and regulate its functions but also it is able to strengthen p53 functions through parallel pathways. For instance, p53 inhibits the kinase mTOR, in response to DNA damage through sestrin1 and 2 activation (35). A recent study has shown that Che-1/AATF inhibits mTOR activity in a p53 independent way, by increasing the transcription of the mTOR inhibitor Redd1 and Deptor in response to different types of cellular stress (36).

## Che-1/AATF Enhances the Oncogenic Potential of Mutant p53 Proteins

As previously described, Che-1/AATF regulates p53 functions in response to DNA damage by increasing its expression and regulating p53 promoter selection. However, very often the proteins involved in p53 activation are also able to sustain and amplify the “gain-of-function” of mtp53 in tumors containing mutated forms of this protein (1). In this regard, Che-1/AATF has shown to play an important role on the activity of the mutant forms of p53. In several breast carcinoma cell lines carrying different forms of mtp53, Che-1/AATF was found accumulated and recruited onto the *TP53* promoter, whereas it was almost undetectable in primary breast epithelial cells (37). According to these findings, Che-1/AATF is required in sustaining mtp53 expression, and its depletion strongly decreases mtp53 expression both at mRNA and protein level, inducing apoptosis without involving any other stimuli. In addition, depletion of Che-1/AATF significantly reduces the expression of important genes involved in DNA repair in cells expressing mtp53, such as BLM and Rad17, inducing in such way endogenous DNA damage and triggering p73 expression as well as its target apoptotic genes *Noxa* and *Puma* (37). It is important to note that, Che-1/AATF depletion did not activate apoptosis in normal cells or in tumor cells carrying either WT-p53 or lacking p53 expression, thereby suggesting that these phenomena require mtp53 downregulation.

It has been widely shown that a major oncogenic ability of the mtp53 proteins is their ability to activate an aberrant transcription of selected target genes involved in cell proliferation by interacting with several transcription factors and being recruited on regulatory regions of chromatin (38). Therefore, it is possible to assume that similar to wild-type p53, this interaction may contribute to aberrant gene regulation conducted by mtp53.

## Che-1/AATF as a Putative Therapeutic Target in Cancer

**Figure 1:**
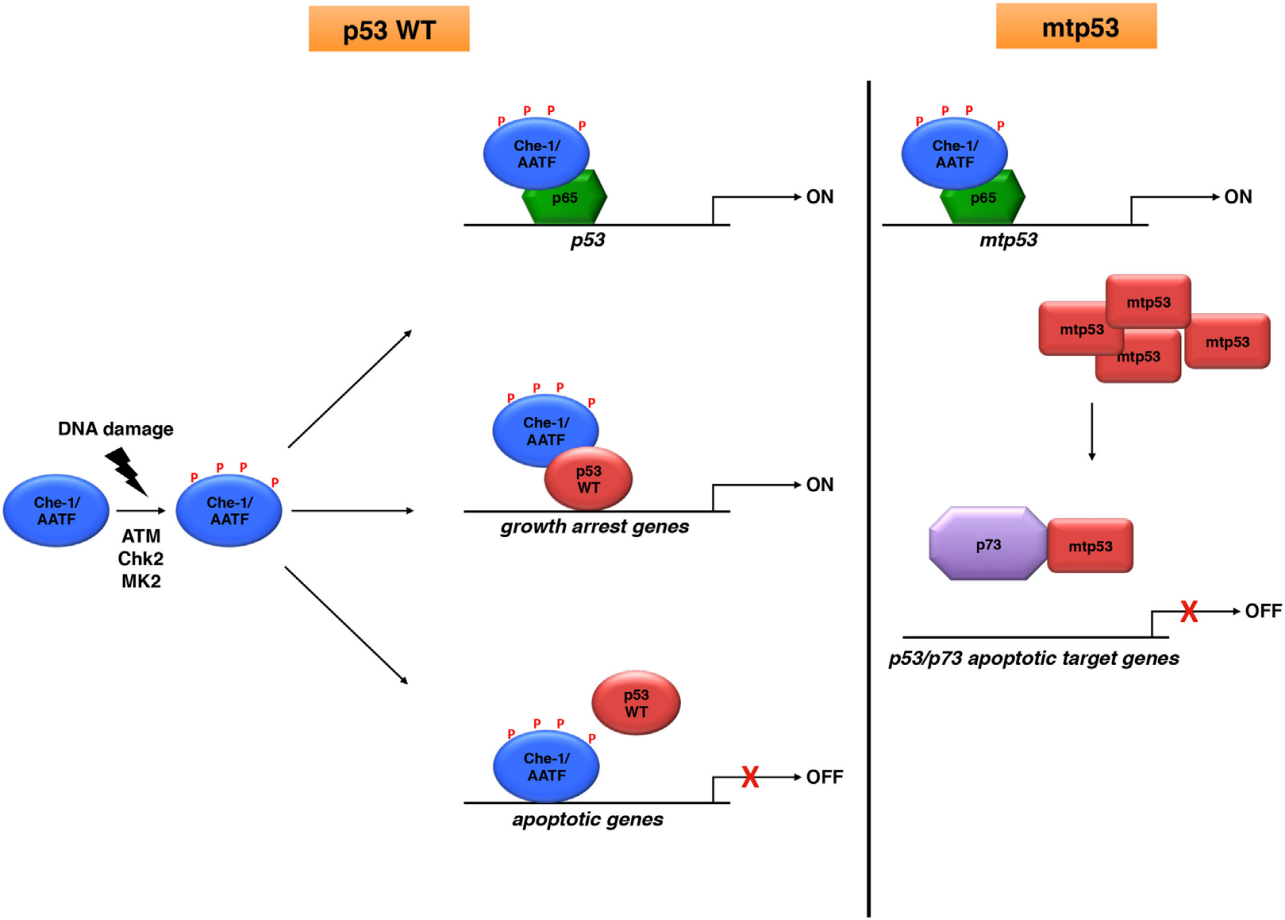
**Che-1/AATF is an endogenous regulator of p53 activities**. Che-1/AATF promotes cell survival in response to DNA damage by regulating both transcription and activity of p53. However, in tumor cells carrying p53 mutations, Che-1/AATF sustains mtp53 levels and promotes p73/mtp53 complex formation, inhibiting in such way the apoptotic activity of p73.

All the observations described above indicate that Che-1/AATF plays a prominent role in many aspects of cancer biology. Even though mutations of Che-1/AATF have not been described so far (39), several studies reported an increase of Che-1/AATF levels in some types in cancer. In particular, elevated levels of this protein have been found in several leukemia cell lines (40) and in patients with chronic lymphocytic leukemia (41) or multiple myeloma (36). In addition, Che-1/AATF gene was found amplified in neuroblastoma patients most of whom expressing wild-type p53, and high levels of Che-1/AATF were found correlated with poor prognosis and reduced survival (32). Importantly, not only this protein is involved in cell cycle progression and in protecting cancer cells from apoptosis induction but also able to control p53 activity (11, 32), inhibiting p53 mediated transcription of apoptotic genes (31, 32). Moreover, Che-1/AATF strongly supports the “gain of function” of the mutated forms of this oncosuppressor (Figure 1) (37). Altogether, these observations indicate that dysregulation of Che-1/AATF expression level could be relevant for the transformation process, and strengthen the notion that Che-1/AATF could be considered a valid target for novel anticancer therapeutic approaches either in tumors expressing wild-type p53, or in cancers carrying its mutated forms. In agreement, Che-1/AATF depletion was shown to increase sensitivity to anticancer agents both *in vitro* and *in vivo* (11, 32, 42), and to activate the apoptotic process in cancer cells carrying mtp53 (37). Unfortunately, no compounds capable of inhibiting Che-1/AATF activity have been identified so far. However, future studies focusing on understanding the mechanisms of action of Che-1/AATF and the characterization of the pathways implicated in its regulation will provide useful indications toward developing specific inhibitors for this protein.

## Author Contributions

TB, SI, and MF equally contributed to write this mini review.

## Conflict of Interest Statement

The authors declare that the research was conducted in the absence of any commercial or financial relationships that could be construed as a potential conflict of interest.

## References

[1] Muller PA Vousden KH p53 mutations in cancer. Nat Cell Biol (2013) 15(1):2–8. 10.1038/ncb2641 23263379

[2] Valenti F Fausti F Biagioni F Shay T Fontemaggi G Domany E Mutant p53 oncogenic functions are sustained by Plk2 kinase through an autoregulatory feedback loop. Cell Cycle (2011) 10(24):4330–40. 10.4161/cc.10.24.18682 22134238

[3] Zerbini LF Wang Y Correa RG Cho JY Libermann TA. Blockage of NF-kappaB induces serine 15 phosphorylation of mutant p53 by JNK kinase in prostate cancer cells. Cell Cycle (2005) 4(9):1247–53. 10.4161/cc.4.9.1966 16082226

[4] Girardini JE Napoli M Piazza S Rustighi A Marotta C Radaelli E A Pin1/mutant p53 axis promotes aggressiveness in breast cancer. Cancer Cell (2011) 20(1):79–91. 10.1016/j.ccr.2011.06.004 21741598

[5] Page G Lödige I Kögel D Scheidtmann KH. AATF, a novel transcription factor that interacts with Dlk/ZIP kinase and interferes with apoptosis. FEBS Lett (1999) 462(1–2):187–91. 10.1016/S0014-5793(99)01529-X 10580117

[6] Fanciulli M Bruno T Di Padova M De Angelis R Iezzi S Iacobini C Identification of a novel partner of RNA polymerase II subunit 11, Che-1, which interacts with and affects the growth suppression function of Rb. FASEB J. (2000) 14(7):904–12. 10783144 10.1096/fasebj.14.7.904

[7] Lindfors K Halttunen T Huotari P Nupponen N Vihinen M Visakorpi T Identification of novel transcription factor-like gene from human intestinal cells. Biochem Biophys Res Commun (2000) 276(2):660–6. 10.1006/bbrc.2000.3480 11027528

[8] Iezzi S Fanciulli M. Discovering Che-1/AATF: a new attractive target for cancer therapy. Front Genet (2015) 6:141. 10.3389/fgene.2015.00141 25914721 PMC4392318

[9] Leister P Burgdorf S Scheidtmann KH Apoptosis antagonizing transcription factor AATF is a novel coactivator of nuclear hormone receptors. Signal Trasduction (2003) 1-2:17–25. 10.1002/sita.200300020

[10] Bruno T De Angelis R De Nicola F Barbato C Di Padova M Corbi N Che-1 affects cell growth by interfering with the recruitment of HDAC1 by Rb. Cancer Cell (2002) 2(5):387–99. 10.1016/S1535-6108(02)00182-4 12450794

[11] Bruno T De Nicola F Iezzi S Lecis D D’Angelo C Di Padova M Che-1 phosphorylation by ATM/ATR and Chk2 kinases activates p53 transcription and the G_2_/M checkpoint. Cancer Cell (2006) 10(6):473–86. 10.1016/j.ccr.2006.10.012 17157788

[12] Ishigaki S Fonseca SG Oslowski CM Jurczyk A Shearstone JR Zhu LJ AATF mediates an antiapoptotic effect of the unfolded protein response through transcriptional regulation of AKT1. Cell Death Differ (2010) 17(5):774–86. 10.1038/cdd.2009.175 19911006 PMC2854298

[13] Grandemange S Schaller S Yamano S Du Manoir S Shpakovski GV Mattei MG A human RNA polymerase II subunit is encoded by a recently generated multigene family. BMC Mol Biol (2001) 2:14. 10.1186/1471-2199-2-14 11747469 PMC61041

[14] Benga WJ Grandemange S Shpakovski GV Shematorova EK Kedinger C Vigneron M. Distinct regions of RPB11 are required for heterodimerization with RPB3 in human and yeast RNA polymerase II. Nucleic Acids Res (2005) 33(11):3582–90. 10.1093/nar/gki672 15987790 PMC1159119

[15] Thomas T Voss AK Petrou P Gruss P The murine gene, Traube, is essential for the growth of preimplantation embryos. Dev Biol. (2000) 227(2):324–42. 10.1006/dbio.2000.9915 11071758

[16] Robert A Margall-Ducos G Guidotti JE Brégerie O Celati C Bréchot C The intraflagellar transport component IFT88/polaris is a centrosomal protein regulating G1-S transition in non-ciliated cells. J Cell Sci (2007) 120(4):628–37. 10.1242/jcs.03422 17264151

[17] Sorino C Bruno T Desantis A Di Certo MG Iezzi S De Nicola F Centrosomal Che-1 protein is involved in the regulation of mitosis and DNA damage response by mediating pericentrin (PCNT)-dependent Chk1 protein localization. J Biol Chem (2013) 288(32):23348–57. 10.1074/jbc.M113.465302 23798705 PMC3743504

[18] Di Certo MG Corbi N Bruno T Iezzi S De Nicola F Desantis A NRAGE associates with the anti-apoptotic factor Che-1 and regulates its degradation to induce cell death. J Cell Sci (2007) 120(11):1852–8. 10.1242/jcs.03454 17488777

[19] Guo Q Xie J. AATF inhibits aberrant production of amyloid beta peptide 1-42 by interacting directly with Par-4. J Biol Chem (2004) 279(6):4596–603. 10.1074/jbc.M309811200 14627703

[20] Xie J Guo Q. AATF protects neural cells against oxidative damage induced by amyloid beta-peptide. Neurobiol Dis (2004) 16(1):150–7. 10.1016/j.nbd.2004.02.003 15207272

[21] Buontempo S Barbato C Bruno T Corbi N Ciotti MT Floridi A Che-1 enhances cyclin-dependent kinase 5 expression and interacts with the active kinase-complex. Neuroreport (2008) 19(5):531–5. 10.1097/WNR.0b013e3282f85c1b 18388733

[22] Barbato C Corbi N Canu N Fanciulli M Serafino A Ciotti M Rb binding protein Che-1 interacts with Tau in cerebellar granule neurons. Modulation during neuronal apoptosis. Mol Cell Neurosci. (2003) 24(4):1038–50. 10.1016/j.mcn.2003.08.002 14697667

[23] Xie J Guo Q. Apoptosis antagonizing transcription factor protects renal tubule cells against oxidative damage and apoptosis induced by ischemia-reperfusion. J Am Soc Nephrol (2006) 17(12):3336–46. 10.1681/ASN.2006040311 17065240

[24] Ferraris SE Isoniemi K Torvaldson E Anckar J Westermarck J Eriksson JE. Nucleolar AATF regulates c-Jun-mediated apoptosis. Mol Biol Cell (2012) 23(21):4323–32. 10.1091/mbc.E12-05-0419 22933572 PMC3484108

[25] Jackson SP Bartek J. The DNA-damage response in human biology and disease. Nature (2009) 461(7267):1071–8. 10.1038/nature08467 19847258 PMC2906700

[26] Lord CJ Ashworth A The DNA damage response and cancer therapy. Nature (2012) 481(7381):287–94. 10.1038/nature10760 22258607

[27] Ciccia A Elledge SJ. The DNA damage response: making it safe to play with knives. Mol Cell (2010) 40(2):179–204. 10.1016/j.molcel.2010.09.019 20965415 PMC2988877

[28] Wang S El-Deiry WS. p73 or p53 directly regulates human p53 transcription to maintain cell cycle checkpoints. Cancer Res (2006) 66(14):6982–9. 10.1158/0008-5472.CAN-06-0511 16849542

[29] Takagi M Absalon MJ McLure KG Kastan MB. Regulation of p53 translation and induction after DNA damage by ribosomal protein L26 and nucleolin. Cell (2005) 123(1):49–63. 10.1016/j.cell.2005.07.034 16213212

[30] Vousden KH Lu X Live or let die: the cell’s response to p53. Nat Rev Cancer (2002) 8:594–604. 10.1038/nrc864 12154352

[31] Desantis A Bruno T Catena V De Nicola F Iezzi S Sorino C Che-1 modulates the decision between cell cycle arrest and apoptosis by its binding to p53. Cell Death Dis. (2015) 6:e1764 10.1038/cddis.2015.117 25996291 PMC4669697

[32] Höpker K Hagmann H Khurshid S Chen S Hasskamp P Seeger-Nukpezah T AATF/Che-1 acts as a phosphorylation-dependent molecular modulator to repress p53-driven apoptosis. EMBO J (2012) 31(20):3961–75. 10.1038/emboj.2012.236 22909821 PMC3474921

[33] De Nicola F Bruno T Iezzi S Di Padova M Floridi A Passananti C The prolyl isomerase Pin1 affects Che-1 stability in response to apoptotic DNA damage. J Biol Chem (2007) 282(27):19685–91. 10.1074/jbc.M610282200 17468107

[34] De Nicola F Catena V Rinaldo C Bruno T Iezzi S Sorino C HIPK2 sustains apoptotic response by phosphorylating Che-1/AATF and promoting its degradation. Cell Death Dis. (2014) 5:e1414. 10.1038/cddis.2014.381 25210797 PMC4225224

[35] Budanov AV Karin M. p53 target genes sestrin1 and sestrin2 connect genotoxic stress and mTOR signaling. Cell (2008) 134(3):451–60. 10.1016/j.cell.2008.06.028 18692468 PMC2758522

[36] Desantis A Bruno T Catena V De Nicola F Goeman F Iezzi S Che-1-induced inhibition of mTOR pathway enables stress-induced autophagy. EMBO J (2015) 34(9):1214–30. 10.15252/embj.201489920 25770584 PMC4426481

[37] Bruno T Desantis A Bossi G Di Agostino S Sorino C De Nicola F Che-1 promotes tumor cell survival by sustaining mutant p53 transcription and inhibiting DNA damage response activation. Cancer Cell (2010) 18(2):122–34. 10.1016/j.ccr.2010.05.027 20708154

[38] Santoro R Strano S Blandino G. Transcriptional regulation by mutant p53 and oncogenesis. Subcell Biochem (2014) 85:91–103. 10.1007/978-94-017-9211-0_5 25201190

[39] Haanpää M Reiman M Nikkilä J Erkko H Pylkäs K Winqvist R. Mutation analysis of the AATF gene in breast cancer families. BMC Cancer (2009) 9:457. 10.1186/1471-2407-9-457 20025740 PMC2806411

[40] Kaul D Mehrotra A. Functional characterization of AATF transcriptome in human leukemic cells. Mol Cell Biochem (2007) 297(1–2):215–20. 10.1007/s11010-006-9317-1 17006618

[41] Bacalini MG Tavolaro S Peragine N Marinelli M Santangelo S Del Giudice I A subset of chronic lymphocytic leukemia patients display reduced levels of PARP1 expression coupled with a defective irradiation-induced apoptosis. Exp Hematol (2012) 40(3):197–206. 10.1016/j.exphem.2011.11.005 22120020

[42] Bruno T Iezzi S De Nicola F Di Padova M Desantis A Scarsella M Che-1 activates XIAP expression in response to DNA damage. Cell Death Differ (2008) 15(3):515–20. 18049476 10.1038/sj.cdd.4402284

